# Relationship Between Gender and the Effectiveness of Montelukast: An Italian/Danish Register-Based Retrospective Cohort Study

**DOI:** 10.3389/fphar.2018.00844

**Published:** 2018-08-02

**Authors:** Maurizio Sessa, Annamaria Mascolo, Bruno D'Agostino, Antonio Casciotta, Vincenzo D'Agostino, Fausto De Michele, Mario Polverino, Giuseppe Spaziano, Mikkel Porsborg Andersen, Kristian Kragholm, Francesco Rossi, Christian Torp-Pedersen, Annalisa Capuano

**Affiliations:** ^1^Department of Drug Design and Pharmacology, University of Copenhagen, Copenhagen, Denmark; ^2^Department of Experimental Medicine, University of Campania “L. Vanvitelli”, Naples, Italy; ^3^Local Health Unit Napoli Second, Department of Pharmaceutical, Naples, Italy; ^4^Department of Pneumology, AORN A. Cardarelli, Naples, Italy; ^5^Department of Pneumology and Endoscopic Unit, Ospedale Scarlato, Scafati, Italy; ^6^Unit of Epidemiology and Biostatistics, Aalborg University Hospital, Aalborg, Denmark; ^7^Department of Cardiology, North Denmark Regional Hospital, Hjørring, Denmark; ^8^Department of Cardiology, Aalborg University Hospital, Aalborg, Denmark; ^9^Department of Health Science and Technology, Aalborg University, Aalborg, Denmark

**Keywords:** clinical epidemiology, asthma, humans, pharmacoepidemiology, pharmacology, translational medical research, montelukast

## Abstract

**Rationale:** Gender-related differences in asthma prevalence, pathophysiology and clinical features induced by sex steroids have been investigated, however, how gender influences response to asthma treatments in routine clinical practice have not yet been elucidated fully. This aspect is crucial for montelukast considering the jeopardization of asthmatic patients that benefit from this treatment and the existence of evidence of gender differences in leukotriene levels. Therefore, to fulfill this medical need, we investigated the role of gender on a set of montelukast' effectiveness surrogates in adults and pediatric patients with asthma.

**Methods:** The study settings were Napoli 2 Local Health Unit (southern Italy) and the entire Danish territory. The study population was composed of adult and pediatric patients with asthma. Cumulative incidence curves, unadjusted and adjusted Cox regression were used as statistical models to compare aforementioned outcomes between genders.

**Results:** Adult Italian male users of montelukast had a statistically lower persistence in montelukast treatment compared to female users. In the adjusted analyses, they had a higher hazard of montelukast' withdrawal (Hazard Ratio [HR] 1.07; 95% Confidence Interval [CI] 1.01–1.14), add-on/switch to a long-term treatment for asthma following montelukast withdrawal (HR 1.72; 95%CI 1.39–2.12), and rescue therapy with short-acting β2 agonist (HR 1.24; 95%CI 1.04–1.47). In the adult Danish cohort, we also found that male users had higher a hazard of rescue therapy with oral corticosteroids (HR 1.10; 95%CI 1.04–1.16). In the pediatric cohorts, no statistically significant differences were observed between genders for aforementioned outcomes.

**Conclusions:** In adults, male gender was associated with increased hazards of montelukast discontinuation, add-on/switch to a long-term treatment for asthma following montelukast withdrawal, and rescue therapy with oral corticosteroids or short-acting β2 agonist when compared to the female gender. As expected, these associations were reversed or absent in pediatric patients.

## Introduction

Gender-related differences in asthma prevalence, pathophysiology and clinical features induced by sex steroids have been investigated, however, how gender influences response to asthma treatments in routine clinical practice have not yet been elucidated fully (Hanley, 1981; Eliasson et al., 1986; Postma, 2007; Pignataro et al., 2017).

In the last decade, new milestones have been set to understand the biological pathways in which sex steroids are involved in determining gender differences in asthma. Pioneering experiments performed by Pergola and colleagues proved that in stimulated whole blood, testosterone and 5α-dihydrotestosterone were able to modulate leukotriene biosynthesis by activating extracellular signal-regulated kinases (ERKs). In turn, ERK represses phospholipase D, causing a reduced biosynthesis of leukotriene by 5-lipoxygenase. These phenomena lead to higher blood concentrations of leukotrienes in women's blood than men (Pergola et al., 2008, 2011).

Additionally, experiments on preclinical models showed an impaired modulation of leukotriene pathway mediated by androgens in male rats and mice that unbalanced the rate of severe asthma exacerbation between the two genders when both were exposed to leukotriene biosynthesis inhibitors. This effect was abolished by administrating blood or leukocytes with 5α-dihydrotestosterone to female mice and rats suggesting a potential gender difference mediated by sex steroids in the effectiveness of the pharmacological treatment with anti-leukotriene between male and female (Pace et al., 2017).

Accordingly, clinical studies investigating the impact of sex steroids in asthma exacerbation during menstrual cycle found that modification of sex steroids levels was associated with a higher concentration of inflammatory biomarkers, including serum leukotriene B4/C4 (Skobeloff et al., 1996; Nakasato et al., 1999; Rossi et al., 2014).

These phenomena might be crucial for montelukast, the most used leukotriene receptor antagonist in several European countries (Sen et al., 2011; Henriksen et al., 2017). Montelukast, in fact, antagonizes the cysteinyl leukotriene receptor CysLT1 in the lungs and bronchial tubes preventing the biological effects mediated by leukotriene D4 (and secondary ligands, leukotrienes C4 and E4) (Paggiaro and Bacci, 2011; Hon et al., 2014).

Understanding if gender is a key predictor for anti-leukotriene treatment' effectiveness is pivotal considering the jeopardization of asthmatic patients that benefit from leukotriene receptor antagonist, whose population, to date, it is not well characterized (Helms, 2000; Borish, 2002; Nayak, 2004; Chauhan et al., 2013, 2017; Bush, 2015; Cingi et al., 2015; Marcello and Carlo, 2016; Pyasi et al., 2016). Moreover, providing new evidence for gender differences in asthma' treatment is needed considering that clinical studies investigating aforementioned associations are missing (Pignataro et al., 2017).

Therefore, given the paucity of evidence on this topic, we investigated the role of gender on a set of montelukast' effectiveness surrogates in adults and pediatric patients with asthma.

## Methods

### Data sources

For the purpose of this study, we used both Napoli Second Local Health Unit (Campania Region, Italy) and Danish administrative databases. Napoli Second Local Health Unit administrative database was set up in December 2014, and currently, contains information on 1,051,883 individuals living in the catchment area of Naples and registered on the lists of 761 general practitioners. Collected information included patients' demographic characteristics, the cause of hospital admission coded according to the International Classification of Disease, 9th revision (ICD-9), and drug prescription classified according to the Anatomical Therapeutic Chemical (ATC) classification system. By using medical diagnoses leading to pharmacological prescriptions and causes of hospital admission, we were able to obtain information on patients' concurrent comorbidities. For each prescription, in fact, general practitioners were required to code medical diagnoses for the aforementioned prescription that was coded according to ICD-9. Therefore, Napoli Second Local Health Unit administrative database allowed us to track anonymously hospital access, clinical and drug history routinely collected for each patient from December 2014 to June 2017. For Danish registries instead, we were able to obtain the same information for the entire Danish population by using the Danish Civil Registration System, the Danish National Causes of Death Registry, the Danish National Patient Registry, and the Danish Registry of Medicinal Product Statistics. The original role and composition of each Danish administrative registry are fully recognized in clinical epidemiology and it is described elsewhere (Gaist et al., 1997; Helweg-Larsen, 2011; Lynge et al., 2011).

### Italian study population

The Italian study population was composed of patients with asthma treated with montelukast for the first time from June 1, 2015 to June 30, 2017. A patient was defined as treated with montelukast for asthma if he/she redeemed a prescription for a drug with an ATC code R03DC03 or R03DC53 and the prescription had 493 (the ICD-9 code) as the cause for montelukast prescription. The date of the first redeeming of such prescription was used as the index date for each patient. In all, two cohorts were retrieved from the Italian population, the Italian pediatric cohort or rather patients with an age <18 years at the first prescription of montelukast and the Italian adult cohort or rather patients with an age ≥18 years at the first prescription of montelukast.

### Danish study population

All patients with a diagnosis of asthma from January 1, 1995 to December 31, 2012 that were treated for the first time with montelukast were identified in the Danish registries. We defined a patient as having a diagnosis of asthma if he/she had primary or secondary hospitalization diagnoses coded as J45 or J46 in accordance with ICD-10 or as ICD-8 codes 493. The diagnosis of asthma has been validated in the Danish Patient Registry (specificity of 98%) (Jensen AØ et al., 2010). A patient was defined as being treated with montelukast if he/she had redeemed prescriptions of a drug with an ATC code R03DC03 or R03DC53. The date of the first redeeming of such prescription was used as the index date for each patient. As for the Italian population, the Danish population was divided in the Danish pediatric cohort and in the Danish adult cohort.

### Follow-up period

The Danish population was followed in administrative registries from the index date to censoring for death or on December 31, 2012 while the Italian population up to June 30, 2017 due to data availability. To estimate the period of exposure to montelukast, the ongoing exposure was calculated for each individual by dividing the number of posological units dispensed by the estimated average dosage for asthma indication as described elsewhere (Andersson et al., 2012).

### Outcomes

The study outcomes were: (1) the withdrawal of montelukast; (2) the add-on or switch to a long-term treatment for asthma following montelukast withdrawal, and (3) the redemption of oral corticosteroids prescription within 12 months from the redemption of the first montelukast prescription. Moreover, (4) the redemption of inhaled short-acting β2 agonist prescription within 12 months from the redemption of the first montelukast prescription. A patient was defined as receiving an add-on or switch to a long-term treatment for asthma if he/she redeemed prescriptions of one or more of the following drugs: inhaled selective β2-adrenoreceptor agonists, inhaled glucocorticoids, mast cell stabilizer, inhaled glucocorticoids + selective β2-adrenoreceptor agonists, phosphodiesterase inhibitors (xanthine derivatives) or mast cell stabilizer + selective β2-adrenoreceptor agonists. ATC codes used to define aforementioned drug classes were provided in Supplementary Table 1.

### Study covariates

Age, gender, year, and month of inclusion, asthma duration, socioeconomic status, comorbidities, and co-treatment were obtained at index date. Comorbidities and co-treatment were evaluated considering all pharmacological prescriptions, hospital access and all medical diagnosis leading to a pharmacological prescription prior or equal to index date. Socioeconomic status was based on family income in the year of inclusion in the cohort and it was divided in quartile. Details on operative definition of comorbidities and co-treatments were provided in Supplementary Tables 2, 3.

### Statistical analyses

At index date, baseline characteristics of men and women were compared using the *t*-tests or ANOVA for continuous variables and χ^2^ or Fisher's exact test for categorical variables. All analyses were based on intention-to-treat approach and used a statistically significant level of *p* < 0.05 (2-sided). In the unadjusted analyses, cumulative incidence curves were generated to compare cumulative incidence for aforementioned study outcomes between genders. Grey's test was used to evaluate the hypotheses that cause-specific cumulative functions were equal for male and females (Austin et al., 2016). Time to event for each study outcome was computed as restricted mean time or median for male and female users of montelukast (Zhao et al., 2016). Additionally, in the unadjusted analysis, Cox regression with only outcome and exposure was used to compare the hazard for aforementioned outcomes between genders. In the adjusted analyses, multivariable Cox regression with aforementioned study covariates was used to evaluate the effect of gender variable upon aforementioned outcomes.

### Compliance with ethical standards

In Italy and Denmark, register-based retrospective studies do not require ethical approval. Patient records/information was anonymized and de-identified prior to data analysis.

## Results

**Figure 1:**
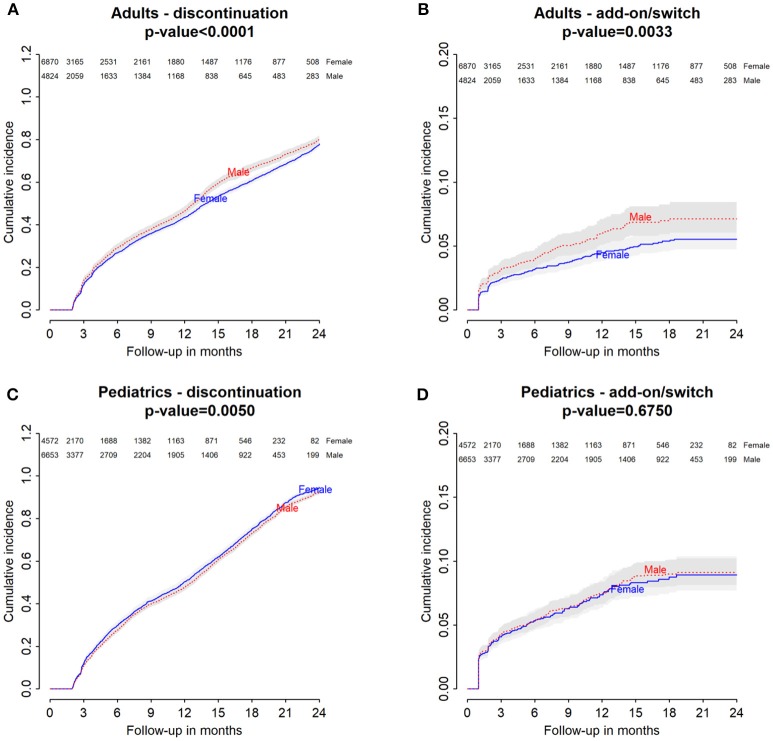
Italian Population. **(A)** Cumulative incidence for montelukast withdrawal—comparison between adult male and female patients. **(B)** Cumulative incidence for an add-on or switch to a long-term treatment for asthma following montelukast withdrawal—comparison between adult male and female patients. **(C)** Cumulative incidence for montelukast withdrawal—comparison between pediatric male and female patients. **(D)** Cumulative incidence for an add-on or switch to a long-term treatment for asthma following montelukast withdrawal—comparison between pediatric male and female patients.

**Figure 2:**
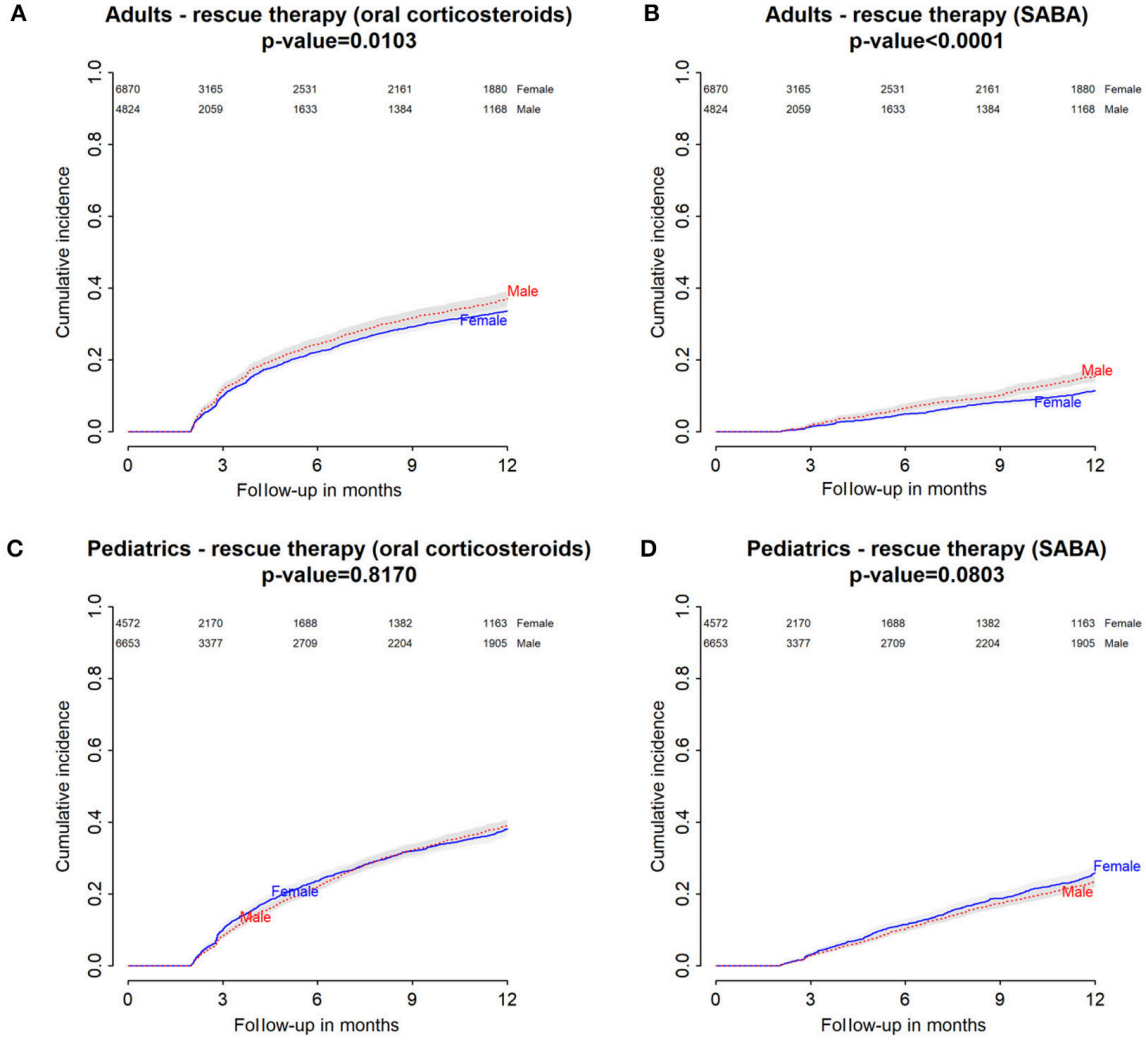
Italian Population. **(A)** Cumulative incidence for rescue therapy with oral corticosteroids among montelukast users—comparison between adult male and female patients. **(B)** Cumulative incidence for rescue therapy with selective β2-adrenoreceptor agonists (SABA) among montelukast users—comparison between adult male and female patients. **(C)** Cumulative incidence for rescue therapy with oral corticosteroids among montelukast users—comparison between pediatric male and female patients. **(D)** Cumulative incidence for rescue therapy with SABA among montelukast users—comparison between pediatric male and female patients.

**Figure 3:**
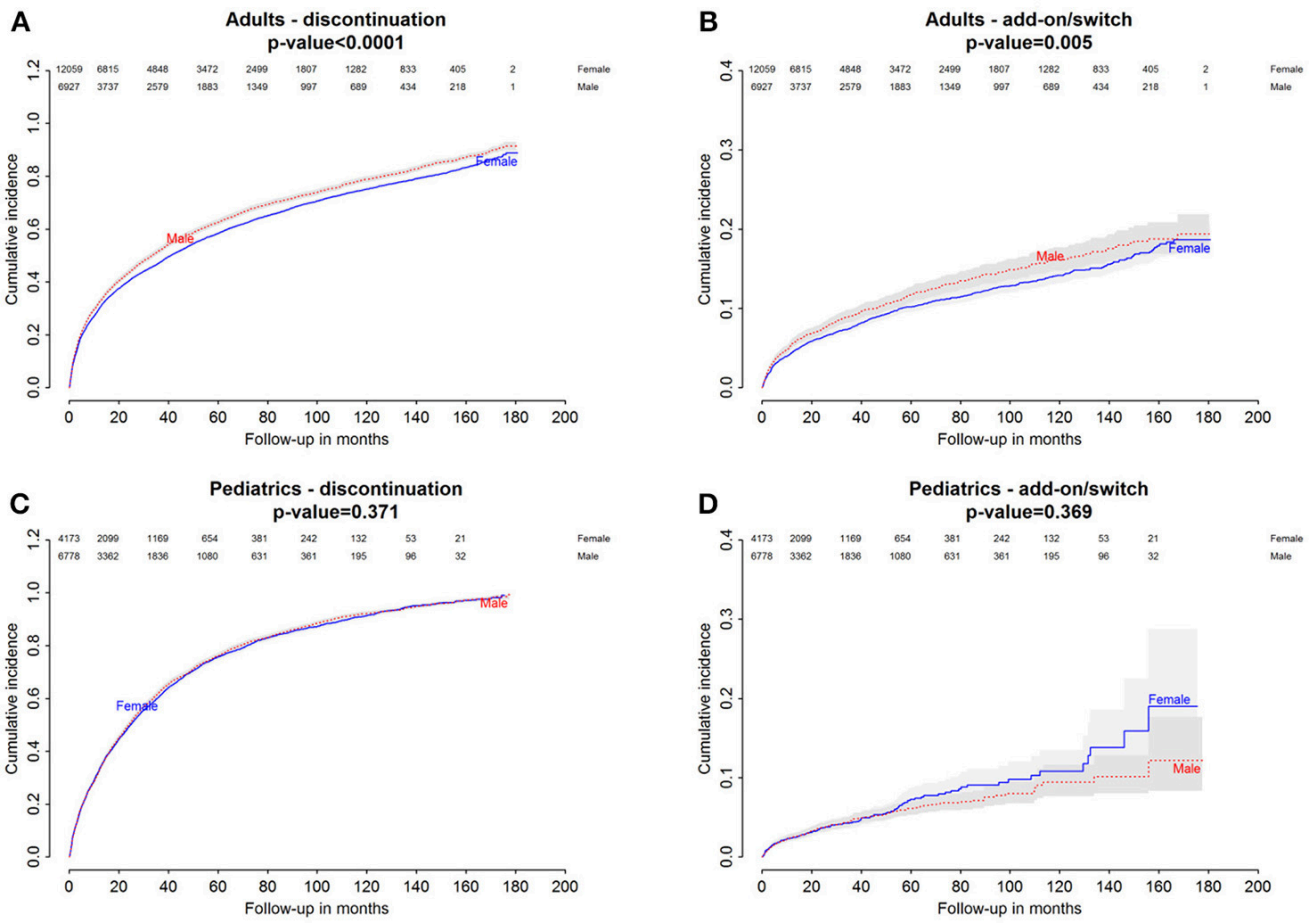
Danish Population. **(A)** Cumulative incidence for montelukast withdrawal—comparison between adult male and female patients. **(B)** Cumulative incidence for an add-on or switch to a long-term treatment for asthma following montelukast withdrawal—comparison between adult male and female patients. **(C)** Cumulative incidence for montelukast withdrawal—comparison between pediatric male and female patients. **(D)** Cumulative incidence for an add-on or switch to a long-term treatment for asthma following montelukast withdrawal—comparison between pediatric male and female patients.

**Figure 4:**
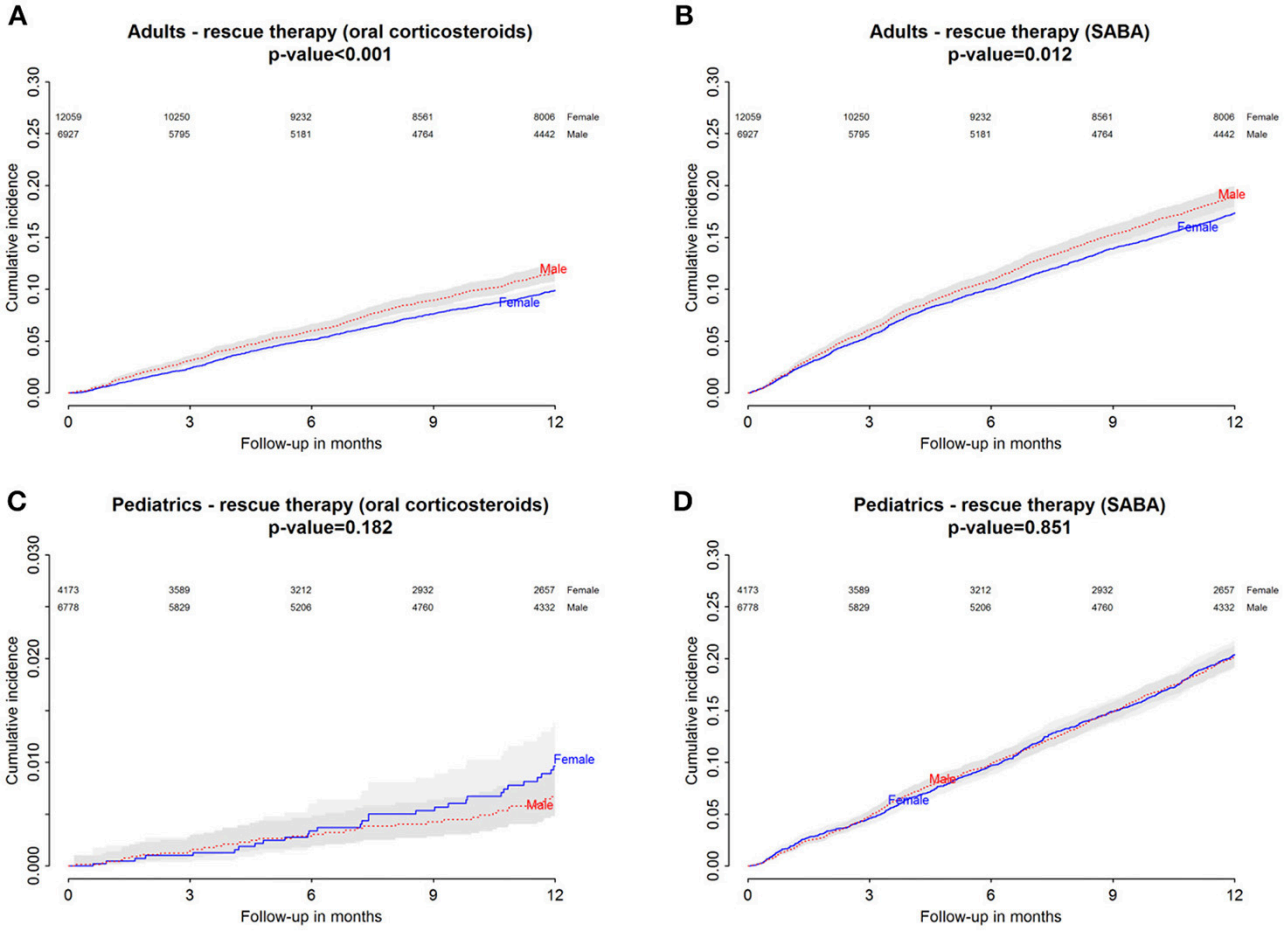
Danish Population. **(A)** Cumulative incidence for rescue therapy with oral corticosteroids among montelukast users—comparison between adult male and female patients. **(B)** Cumulative incidence for rescue therapy with selective β2-adrenoreceptor agonists (SABA) among montelukast users—comparison between adult male and female patients. **(C)** Cumulative incidence for rescue therapy with oral corticosteroids among montelukast users—comparison between pediatric male and female patients. **(D)** Cumulative incidence for rescue therapy with SABA among montelukast users-comparison between pediatric male and female patients.

Baseline characteristics of adults and pediatric patients are provided in Tables 1, 2, respectively. Cause-specific cumulative incidence curves for male and female users of montelukast in the Italian/Danish cohorts are provided in Figures 1–4.

**Table 1:** Baseline characteristics of Italian/Danish adult patients with asthma at the first prescription of montelukast.

	**Danish Population**					**Italian Population**			
**Variable**	**Level**	**Female (*n* = 12,059)**	**Male (*n* = 6,927)**	**Total (*n* = 18,986)**	***p*-value**	**Female (*n* = 6,870)**	**Male (*n* = 4,824)**	**Total (*n* = 11,694)**	***p*-value**
Socio Economic Status	Quartile 1	3222 (26.7)	1525 (22.0)	4747 (25.0)		-	-	-	
	Quartile 2	3098 (25.7)	1649 (23.8)	4747 (25.0)		-	-	-	
	Quartile 3	2918 (24.2)	1828 (26.4)	4746 (25.0)		-	-	-	
	Quartile 4	2821 (23.4)	1925 (27.8)	4746 (25.0)	<0.001	-	-	-	
Age	mean (SD)	50.6 (16.8)	50.1 (16.8)	50.5 (16.8)	0.048	53.5 (21.9)	53.1 (26.6)	53.3 (24.0)	0.384
Asthma duration	mean (SD)	6.6 (7.6)	7.4 (8.0)	6.9 (7.7)	<0.001	-	-	-	
Heart failure	Yes	1090 (9.0)	788 (11.4)	1878 (9.9)	<0.001	611(8.9)	555 (11.5)	1166 (10.0)	<0.001
Chronic kidney disease	Yes	372 (3.1)	275 (4.0)	647 (3.4)	0.001	227 (3.3)	217 (4.5)	444 (3.8)	<0.001
β-blockers	Yes	510 (4.2)	262(3.8)	772 (4.1)	0.143	1300 (18.9)	656 (13.6)	1956 (16.7)	<0.001
Nonsteroidal anti-inflammatory drug	Yes	1362 (11.3)	556 (8.0)	1918 (10.1)	<0.001	1067 (15.5)	454 (9.4)	1521 (13.0)	<0.001
Antibiotics-systemic	Yes	3704 (30.7)	1695 (24.5)	5399 (28.4)	<0.001	2882 (42.0)	1775 (36.8)	4657 (39.8)	<0.001
Low-dose acetylsalicylic acid	Yes	568 (4.7)	337 (4.9)	905 (4.8)	0.655	364 (5.3)	214 (4.4)	578 (4.9)	0.038
β2 agonist short acting-inhalers	Yes	8397 (69.6)	4982 (71.9)	13379 (70.5)	<0.001	2169 (31.6)	1576 (32.7)	3745 (32.0)	0.218
β2 agonist short acting-oral	Yes	853 (7.1)	501 (7.2)	1354 (7.1)	0.703	0 (0.0)	0 (0.0)	0 (0.0)	1.000
β2 agonist long acting-inhalers	Yes	4343 (36.0)	2371 (34.2)	6714 (35.4)	0.014	497 (7.2)	292 (6.1)	789 (6.7)	0.013
β2 agonist long acting-oral	Yes	182 (1.6)	113 (1.6)	300 (1.6)	0.712	0 (0.0)	0 (0.0)	0 (0.0)	1.000
Glucorticoids	Yes	8008 (66.4)	4561 (65.8)	12569 (66.2)	0.439	4035 (58.7)	2649 (54.9)	6684 (57.2)	<0.001
Glucorticoid + β2 agonist	Yes	4571 (37.9)	2673 (38.6)	7244 (38.2)	0.359	3843 (55.9)	2189 (45.4)	6032 (51.6)	<0.001
β2 agonist (non-selective)	Yes	5 (0.0)	4 (0.1)	9 (0.0)	0.880	0 (0.0)	0 (0.0)	0 (0.0)	1.000
Mast-cell stabilizer	Yes	44 (0.4)	36 (0.5)	80 (0.4)	0.142	111 (1.6)	50 (1.0)	161 (1.4)	0.010

**Table 2:** Baseline characteristics of Italian/Danish pediatric patients with asthma at the first prescription of montelukast.

	**Danish Population**					**Italian Population**			
**Variable**	**Level**	**Female (*n* = 4,173)**	**Male (*n* = 6,778)**	**Total (*n* = 10,951)**	***p*-value**	**Female (*n* = 4,572)**	**Male (*n* = 6,653)**	**Total (*n* = 11,225)**	***p*-value**
Socio Economic Status	Quartile 1	830 (19.9)	1425 (21.0)	2255 (20.6)		-	-	-	
	Quartile 2	429 (10.3)	535 (7.9)	964 (8.8)		-	-	-	
	Quartile 3	607 (14.5)	907 (13.4)	1514 (13.8)		-	-	-	
	Quartile 4	754 (18.1)	953 (14.1)	1707 (15.6)		-	-	-	
	Missing	1553 (37.2)	2958 (43.6)	4511 (41.2)	<0.001	-	-	-	
Age	mean (SD)	8.2 (5.2)	7.1 (4.8)	7.5 (5.0)	<0.001	6.8 (4.1)	7.2 (4.3)	7.0 (4.2)	<0.001
Asthma duration	mean (SD)	3.2 (3.7)	3.3 (3.6)	3.3 (3.6)	0.234	-	-	-	
Nonsteroidal anti-inflammatory drug	Yes	39 (0.9)	21 (0.3)	60 (0.5)	<0.001	1067 (15.5)	454 (9.4)	1521 (13.0)	<0.001
Antibiotics-systemic	Yes	832 (19.9)	1365 (20.1)	2197 (20.1)	0.818	2882 (42.0)	1775 (36.8)	4675 (39.8)	<0.001
β2 agonist short acting-inhalers	Yes	3209 (76.9)	5326 (78.6)	8535 (77.9)	0.042	2756 (60.3)	4152 (62.4)	6908 (61.5)	0.024
β2 agonist short acting-oral	Yes	211 (5.1)	395 (5.8)	606 (5.5)	0.095	0 (0.0)	0 (0.0)	0 (0.0)	1.000
β2 agonist long acting-inhalers	Yes	566 (13.6)	768 (11.3)	1334 (12.2)	<0.001	11 (0.2)	13 (0.2)	24 (0.2)	0.763
β2 agonist long acting-oral	Yes	0 (0.0)	0 (0.0)	0 (0.0)	1.000	0 (0.0)	0 (0.0)	0 (0.0)	1.000
Glucorticoids	Yes	3191 (76.5)	5370 (79.2)	8561 (78.2)	<0.001	3908 (85.5)	5636 (84.7)	9544 (85.0)	0.277
Glucorticoid + β2 agonist	Yes	728 (17.4)	1051 (15.5)	1779 (16.2)	<0.001	586 (12.8)	1054 (15.8)	1640 (14.6)	<0.001
β2 agonist (non-selective)	Yes	0 (0.0)	0 (0.0)	0 (0.0)	1.000	0 (0.0)	0 (0.0)	0 (0.0)	1.000
Mast-cell stabilizer	Yes	3 (0.1)	7 (0.1)	10 (0.1)	0.894	69 (1.5)	113 (1.7)	182 (1.6)	0.481

### Gender differences in the hazard of montelukast withdrawal

Italian and Danish male users had a statistically lower persistence in montelukast treatment compared to female users and a higher hazard of montelukast' withdrawal. In the pediatric Italian cohort, the association was reversed. In fact, male users had a higher persistence in montelukast treatment if compared to female users and a lower hazard of montelukast' withdrawal. In the pediatric Danish population, instead, no statistically significant differences were observed between male and female users of montelukast (Tables 3, 4).

**Table 3:** Comparison between male and female users of montelukast for: (1) persistence in montelukast treatment; (2) restricted mean time free from add-on or switch to a long-term treatment for asthma; (3) restricted mean time free from rescue therapy with oral corticosteroids and (4) restricted mean time free from rescue therapy with rescue therapy with short-acting β2 agonists.

**Cohorts**	**Estimates in months**
**PERSISTENCE IN MONTELUKAST TREATMENT**	
Italian–male-adults	Median 12.80; 95%CI 12.30–13.30
Italian–female-adults	Median 13.87; 95%CI 13.37–14.47
Danish–male-adults	Median 33.37; 95%CI 31.27–35.27
Danish–female-adults	Median 40.77; 95%CI 39.2–42.70
Italian–male-pediatrics	Median 12.57; 95%CI 11.47–12.53
Italian–female-pediatrics	Median 11.90; 95%CI 11.47–12.53
Danish–male-pediatrics	Median 23.90; 95%CI 22.87–25.00
Danish–female-pediatrics	Median 24.73; 95%CI 23.33–26.10
**RESTRICTED MEAN TIME FREE FROM ADD-ON OR SWITCH TO A LONG-TERM TREATMENT FOR ASTHMA**	
Italian–male-adults	Mean 22.7; standard error 0.09
Italian–female-adults	Mean 23.0; standard error 0.07
Danish–male-adults	Mean 172; standard error 1.15
Danish–female-adults	Mean 175; standard error 0.83
Italian–male-pediatrics	Mean 22.7; standard error 0.10
Italian–female-pediatrics	Mean 23.0; standard error 0.07
Danish–male-pediatrics	Mean 169; standard error 0.78
Danish–female-pediatrics	Mean 172; standard error 0.55
**RESTRICTED MEAN TIME FREE FROM RESCUE THERAPY WITH ORAL CORTICOSTEROIDS**	
Italian–male-adults	Mean 9.48; standard error 0.08
Italian–female-adults	Mean 9.70; standard error 0.06
Danish–male-adults	Mean 11.30; standard error 0.03
Danish–female-adults	Mean 11.40; standard error 0.02
Italian–male-pediatrics	Mean 9.58; standard error 0.06
Italian–female-pediatrics	Mean 9.52; standard error 0.07
Danish–male-pediatrics	Mean 11.8; standard error 0.03
Danish–female-pediatrics	Mean 11.7; standard error 0.05
**RESTRICTED MEAN TIME FREE FROM RESCUE THERAPY WITH RESCUE THERAPY WITH SHORT-ACTING** β**2 AGONISTS**	
Italian–male-adults	Mean 11.20; standard error 0.05
Italian–female-adults	Mean 11.40; standard error 0.03
Danish–male-adults	Mean 11.30; standard error 0.03
Danish–female-adults	Mean 11.40; standard error 0.02
Italian–male-pediatrics	Mean 10.80; standard error 0.05
Italian–female-pediatrics	Mean 10.60; standard error 0.06
Danish–male-pediatrics	Mean 7.37; standard error 0.09
Danish–female-pediatrics	Mean 7.40; standard error 0.10

**Table 4:** Unadjusted and adjusted hazard ratio (HR) of: (1) montelukast' withdrawal; (2) add-on or switch to a long-term treatment for asthma; (3) rescue therapies with oral corticosteroids within 12 months from the redemption of the first montelukast prescription and (4) rescue therapies with short-acting β2 agonist within 12 months from the redemption of the first montelukast prescription.

**Cohort**	**Unadjusted-hazard ratio of montelukast' withdrawal**	**Adjusted-hazard ratio of montelukast' withdrawal**	**Unadjusted-hazard ratio of add-on or switch to a long-term treatment for asthma**	**Adjusted-hazard ratio of add-on or switch to a long-term treatment for asthma**	**Unadjusted-hazard ratio of rescue therapies with oral corticosteroids within 12 months from the redemption of the first montelukast prescription**	**Adjusted-hazard ratio of rescue therapies with oral corticosteroids within 12 months from the redemption of the first montelukast prescription**	**Unadjusted-hazard ratio of rescue therapies with short-acting β2 agonist within 12 months from the redemption of the first montelukast prescription**	**Adjusted-hazard ratio of rescue therapies with short-acting β2 agonist within 12 months from the redemption of the first montelukast prescription**
Italian–male vs. female-adults	HR 1.11; 95% Confidence Interval [CI] 1.04–1.17	HR 1.07; 95%CI 1.01–1.14	HR 1.36; 95%CI 1.11–1.67	HR 1.72; 95%CI 1.39–2.12	HR 1.12; 95%CI 1.03–1.23	HR 1.04; 95%CI 0.95–1.14	HR 1.38; 95%CI 1.17–1.63	HR 1.24; 95%CI 1.04–1.47
Danish–male vs. female-adults	HR 1.12; 95%CI 1.08–1.16	HR 1.15; 95%CI 1.10–1.19	HR 1.16; 95%CI 1.05–1.28	HR 1.15; 95%CI 1.04–1.29	HR 1.06; 95%CI 1.01–1.11	HR 1.10; 95%CI 1.04–1.16	HR 1.10; 95%CI 1.04–1.17	HR 1.15; 95%CI 1.08–1.23
Italian–male vs. female-pediatrics	HR 0.93; 95%CI 0.88–0.98	HR 0.94; 95%CI 0.89–0.99	HR 1.04; 95%CI 0.88–1.23	HR 1.05; 95%CI 0.89–1.24	HR 1.01; 95%CI 0.92–1.11	HR 1.02; 95%CI 0.93–1.11	HR 0.90; 95%CI 0.80–1.01	HR 0.93; 95%CI 0.83–1.05
Danish–male vs. female-pediatrics	HR 1.02; 95%CI 0.97–1.06	HR 1.04; 95%CI 0.99–1.08	HR 0.90; 95%CI 0.75–1.08	HR 0.95; 95%CI 0.78–1.15	HR 1.00; 95%CI 0.94–1.07	HR 1.04; 95%CI 0.98–1.09	HR 1.00; 95%CI 0.91–1.09	HR 1.02; 95%CI 0.93–1.12

*vs, versus; female (reference group)*.

### Gender differences in the hazard of add-on/switch of/to a long-term treatment for asthma following montelukast withdrawal

In both Italian and Danish cohorts, male users of montelukast had a lower restricted mean time free from add-on or switch to a long-term treatment for asthma following montelukast withdrawal compared to women and a higher hazard for this outcome (Tables 3, 4). No statistically significant differences were observed between male and female for pediatric cohorts. Add-on or switch performed in Italian and Danish populations are provided in Table 5.

**Table 5:** Drugs added or switched following montelukast withdrawal.

		**Danish Population**				**Italian Population**			
**ATC^‡^ code**	**Active ingredients**	**Adults**		**Pediatrics**		**Adults**		**Pediatrics**	
		**Add-on *N^†^* = 1395**	**Switch *N^†^* = 513**	**Add-on *N^†^* = 296**	**Switch *N^†^* = 173**	**Add-on *N^†^* = 380**	**Switch N^†^ = 270**	**Add-on *N^†^* = 264**	**Switch *N^†^* = 390**
R03AC12	salmeterol	99 (7.1)	23 (4.48)	13 (4.39)		1 (0.26)			
R03AC13	formoterol	167 (12.00)	45 (8.77)	53 (17.94)	17 (9.82)	8 (2.1)	4 (1.49)		
R03AK	adrenergic + corticosteroid					9 (2.36)	5 (1.86)	3 (1.16)	3 (0.76)
R03AK04	salbutamol + cromoglicate	26 (1.86)	7 (1.36)						
R03AK06	salmeterol + fluticasone	200 (14.34)	99 (19.29)	21 (7.09)	26 (15.05)	33 (8.75)	21 (7.78)	7 (2.66)	8 (2.06)
R03AK07	formoterol + budesonide	260 (18.64)	124 (24.22)	47 (15.87)	49 (28.32)	8 (2.1)	13 (4.82)	1 (0.387)	6 (1.54)
R03AK08	formoterol + beclometasone					35 (9.21)	25 (9.26)	5 (1.89)	2 (0.52)
R03AK11	formoterol + fluticasone					15 (3.94)	4 (1.49)		3 (0.77)
R03BA01	beclometasone	29 (2.07)	21 (4.09)	26 (8.78)	10 (5.78)	117 (30.78)	106 (39.26)	91 (34.46)	140 (35.91)
R03BA02	budesonide	317 (22.72)	104 (20.27)	75 (25.33)	33 (19.07)	30 (7.89)	25 (9.25)	53 (20.07)	103 (26.42)
R03BA03	flunisolide					35 (9.21)	22 (8.14)	32 (12.12)	31 (7.95)
R03BA05	fluticasone	87 (6.23)	35 (6.82)	41 (13.85)	38 (21.96)	32 (8.42)	22 (8.14)	70 (26.51)	74 (18.97)
R03BA08	ciclesonide	1 (0.07)				4 (1.05)			1 (0.25)
R03BC01	cromoglicic acid	2 (0.14)							
R03BC03	nedocromil	1 (0.07)							
R03CC12	bambuterol	12 (0.86)	8 (1.55)						3 (0.76)
R03DA04	theophylline	91 (6.52)	24 (4.67)	7 (2.36)		22 (5.78)	8 (2.96)		
R03DA05	aminophylline	4 (0.28)							8 (2.05)
R03DA11	doxofylline					30 (7.89)	13 (4.81)	2 (0.75)	6 (1.53)
R03DC01	zafirlukast	99 (7.1)	23 (4.48)	13 (4.39)		1 (0.26)	2 (0.74)		2 (0.51)

† N, number of add-on or switch;

‡ *ATC, anatomical therapeutic classification*.

### Gender differences in the hazard of receiving rescue therapies with oral corticosteroids within 12 months from the redemption of the first montelukast prescription

Within 12 months from the redemption of the first montelukast prescription, Italian male users had a lower restricted mean time free from rescue therapy with oral corticosteroids compared to female users and a higher hazard for this outcome in the unadjusted analysis but not in the adjusted analysis. Danish male users of montelukast, instead, had a lower restricted mean time free from rescue therapy with oral corticosteroids if compared to female users and a higher hazard for this outcome. In the Italian and Danish pediatric cohorts, no statistically significant differences were observed between male and female users of montelukast (Tables 3, 4).

### Gender differences in the hazard of receiving rescue therapies with short-acting β2 agonist within 12 months from the redemption of the first montelukast prescription

Within 12 months from the redemption of the first montelukast prescription, Italian and Danish male users had a lower restricted mean time free from rescue therapy with short-acting β2 agonist and compared to female users and a higher hazard for this outcome. For pediatric cohorts, no statistically significant differences were observed between Italian and Danish male and female users of montelukast (Tables 3, 4).

## Discussion

To our knowledge, this is the first study providing evidence of gender differences in the persistence with montelukast treatment, in the probability of receiving an add-on/switch of/to a long-term treatment for asthma, and in the probability of receiving a rescue therapy with oral corticosteroids or short-acting β2 agonist in adult patients with asthma.

A plausible explanation for our results is that for women in fertile age, the role of leukotriene for asthma exacerbation is of higher relevance than in male (Hanley, 1981; Eliasson et al., 1986; Skobeloff et al., 1996; Nakasato et al., 1999). This biological difference could have led women to have a higher benefit from the anti-leukotriene therapy in terms of relieving asthma' sign and symptoms resulting in a more favorable prognosis. We hypothesized that the observed gender differences were mediated by sex steroids (Hanley, 1981; Eliasson et al., 1986; Skobeloff et al., 1996; Nakasato et al., 1999).

In line with this hypothesis, it should be emphasized that women in the fertile age, when compared to men, tend to have especially during the preovulatory phase (Osborne et al., 1998; Cydulka et al., 2001; Brenner et al., 2005; Ostrom, 2006; Pereira Vega et al., 2010; Graziottin and Zanello, 2015; Graziottin and Serafini, 2016) a different perception of their symptoms which lead to a poorer control of asthma and to an increase in asthma treatments and healthcare use (Pignataro et al., 2017). Specifically, during preovulatory and perimenstrual phases when serum estradiol levels decrease sharply after the prolonged peak, there is a higher concentration of serum leukotriene C4 levels which is associated with a higher risk of asthma exacerbation (Skobeloff et al., 1996; Nakasato et al., 1999; Green et al., 2004; Brenner et al., 2005; Matteis et al., 2014). We believed, therefore, that when the impact of leukotriene was minimized by using a leukotriene receptor antagonist, montelukast, women were more prone to perceive the benefit of asthma medicines and therefore persist more in treatment and request less add-on/switch or rescue therapies than men.

We also believe that androgens could have played a key role in gender differences for our study outcomes. In accordance with our hypothesis, Pergola and colleagues proved that testosterone and 5α-dihydrotestosterone were able to modulate leukotriene biosynthesis by stimulating ERK which in turn represses phospholipase D causing a reduced biosynthesis of leukotriene by 5-lipoxygenase (Pergola et al., 2008, 2011). Additionally, evidence from preclinical models shows an impaired modulation of leukotriene pathway mediated by androgens in male rats and mice which unbalanced the rate of severe asthma exacerbation between the two genders (Pace et al., 2017). In particular, androgens by modulating the assembly of the complex leukotriene-biosynthetic-5-lipoxygenase/5-lipoxygenase–activating protein required a higher dosage of an inhibitor of 5-lipoxygenase–activating protein which resulted in a more efficient reduction of leukotriene B4 levels in female mice and rats' lung exudates when compared to male (Pace et al., 2017). We believe that if aforementioned effects are directly translatable in humans, male during the fertile age could have benefitted less from an anti-leukotriene therapy given the impact androgens on leukotriene pathway.

In accordance with aforementioned speculations on the involvement of sex hormones for our results, we found that in the Danish prepubertal pediatric cohort, there were no statistically significant differences between male and female users of montelukast for primary and secondary outcomes. In the Italian prepubertal cohort instead, for our study outcomes, associations were reversed or not statistically significant. In this regard, it should be highlighted that as mentioned, both Danish and Italian pediatric cohorts had a mean age that is compatible with a prepubertal phase in which the level of sex steroids are notoriously low (Swerdloff and Odell, 1975; Korth-Schutz et al., 1976; Garnett et al., 2004; Courant et al., 2010; Albin et al., 2012). These results are in line with those published by Johnston et al., which evidenced in a randomized clinical trial that montelukast addition was beneficial in pre-pubertal boys and post-pubertal girls with asthma. In particular, asthmatic boys aged 2–5 years showed greater benefit from montelukast addition if compared to boys aged 0- to 14-year-olds; for girls instead, the beneficial effect of montelukast addition was most evident in 10- to 14-year-olds (Johnston et al., 2007).

We hypothesized, therefore, that in virtue of aforementioned mechanisms, testosterone could have influenced the chance of montelukast of properly relieve sign and symptoms of asthma in male patients, and these phenomena could have resulted in a lower probability of have a positive prognosis for our study outcomes.

## Strengths and limitations

The major strength of this study is the confirmation of results in two different European countries while the main limitation of this study is its observational design, which does not exclude the possibility of unmeasured confounders limiting the estimation of causal effects. Limitations should include the lack of data availability on hormone levels and lack of statistical power to split cohorts into different age groups. Additionally, it should be emphasized that the use of surrogate outcomes and registries to investigate safety-related outcomes have intrinsic limitations that have been described elsewhere (Boissel et al., 1992; Aronson, 2012; Thygesen and Ersbøll, 2014).

## Conclusion

In Italian and Danish adults, male gender was associated with an increased hazard of montelukast discontinuation, add-on/switch to a long-term treatment for asthma following montelukast withdrawal, and rescue therapy with oral corticosteroids or short-acting β2 agonist when compared to the female gender. These differences were lost or reversed in the pediatric population. Despite more studies are necessary to clarify these associations, our results could inspire researchers to investigate further the beneficial effect of a possible gender-oriented pharmacological treatment of asthma. In order to overcome the limitations of this study we are now conducting a prospective cohort study in collaboration with the Respiratory Department of the “Mauro Scarlato” Hospital (Scafati, Italy).

## Datasets are available on request

The raw data supporting the conclusions of this manuscript will be made available by the authors, with legal reservations.

## Author contributions

BD, MS, CT-P, AM, ACap, and FR developed the concept and designed the study. MS, AM, ACas, ACap, FR, BD, MP, and CT-P: analysis or interpretation of data. MS, GS, ACas, ACap, VD, FM, MP, MA, KK, FR and CT-P drafting the paper and revising it for important intellectual content. MS, AM, ACap, BD, MP, and CT-P: wrote the paper. MS, GS, ACas, VD, FM, MP, MA, KK, FR, ACap and CT-P: final approval of the version to be published.

### Conflict of interest statement

The authors declare that the research was conducted in the absence of any commercial or financial relationships that could be construed as a potential conflict of interest.
