# Supplementary material for: Relationship Between Gender and the Effectiveness of Montelukast: An Italian/Danish Register-Based Retrospective Cohort Study
Source: Front Pharmacol. 2018 Aug 2;9:844. doi: 10.3389/fphar.2018.00844 (PMC6083053; doi:10.3389/fphar.2018.00844)
Supplement: Supplementary file 1 [file Table_1.DOCX]

Supplementary Material

Relationship between gender and the effectiveness of montelukast: an Italian/Danish register-based retrospective cohort study

Maurizio Sessa^1,2^⸸ & Annamaria Mascolo^2^⸸, Bruno D’Agostino^2^, Antonio Casciotta^3^, Vincenzo D’Agostino^3^, Fausto De Michele^4^, Mario Polverino^5^, Giuseppe Spaziano^2^, Mikkel Porsborg Andersen^6^, Kristian Kragholm^6^, Francesco Rossi^2^, Christian Torp-Pedersen^6,7^ and Annalisa Capuano^2^.

^1^Department of Drug Design and Pharmacology, University of Copenhagen, Copenhagen, Denmark
^2^Department of Experimental Medicine, University of Campania “L. Vanvitelli”, Naples, Italy.
^3^Pharmaceutical Department, Local Health Unit Napoli Second, Napoli, Italy.
^4^Department of Pneumology, AORN A. Cardarelli, Naples, Italy.
^5^Department of Pneumology and Endoscopic Unit, Ospedale Scarlato, Scafati, Italy.
^6^Unit of Epidemiology and Biostatistics, Aalborg University Hospital, Aalborg, Denmark
^7^Department of Health Science and Technology, Aalborg University, Aalborg, Denmark.

⸸ These authors contributed equally and served as co-first authors.

*** Correspondence:**Maurizio Sessa
maurizio.sessa@sund.ku.dk

**Keywords: clinical epidemiology_1_; asthma_2_; humans_3_; pharmacoepidemiology_4_; pharmacology_5_; translational medical research_6_; montelukast_7_**

**Supplementary table 1**. Operative definition of outcomes.

| **Drugs** | **ATC^†^ code** |
| --- | --- |
| *Long-term treatment for asthma*:  selective beta-2-adrenoreceptor agonists inhalants and oral – long acting, glucocorticoids, mast cell stabilizer, glucocorticoids + selective beta-2-adrenoreceptor agonists inhalants, and mast cell stabilizer + selective beta-2-adrenoreceptor agonists inhalants | R03AC18, R03AC13, R03AC12,R03CC12, R03BA01, R03BA05, R03BA02, R03BA08, R03BA03, R01AC07, R03BC03, R03BC01, R01AC01, R03AK07, R03AK06, R03AK04, R01AC51 |
| *Short acting selective beta-2-adrenoreceptor agonists* | R03AC04, R03AC05, R03AC03, R03AC02, R03CC03, R03CC02 |
| *Oral corticosteroids* | H02AB |

**^†^**ATC: Anatomical Therapeutic Classification
