# Supplementary material for: Relationship Between Gender and the Effectiveness of Montelukast: An Italian/Danish Register-Based Retrospective Cohort Study
Source: Front Pharmacol. 2018 Aug 2;9:844. doi: 10.3389/fphar.2018.00844 (PMC6083053; doi:10.3389/fphar.2018.00844)
Supplement: Supplementary file 2 [file Table_2.DOCX]

Supplementary Material

Relationship between gender and the effectiveness of montelukast: an Italian/Danish register-based retrospective cohort study

Maurizio Sessa^1,2^⸸ & Annamaria Mascolo^2^⸸, Bruno D’Agostino^2^, Antonio Casciotta^3^, Vincenzo D’Agostino^3^, Fausto De Michele^4^, Mario Polverino^5^, Giuseppe Spaziano^2^, Mikkel Porsborg Andersen^6^, Kristian Kragholm^6^, Francesco Rossi^2^, Christian Torp-Pedersen^6,7^ and Annalisa Capuano^2^.

^1^Department of Drug Design and Pharmacology, University of Copenhagen, Copenhagen, Denmark
^2^Department of Experimental Medicine, University of Campania “L. Vanvitelli”, Naples, Italy.
^3^Pharmaceutical Department, Local Health Unit Napoli Second, Napoli, Italy.
^4^Department of Pneumology, AORN A. Cardarelli, Naples, Italy.
^5^Department of Pneumology and Endoscopic Unit, Ospedale Scarlato, Scafati, Italy.
^6^Unit of Epidemiology and Biostatistics, Aalborg University Hospital, Aalborg, Denmark
^7^Department of Health Science and Technology, Aalborg University, Aalborg, Denmark.

⸸ These authors contributed equally and served as co-first authors.

*** Correspondence:**Maurizio Sessa
maurizio.sessa@sund.ku.dk

**Keywords: clinical epidemiology_1_; asthma_2_; humans_3_; pharmacoepidemiology_4_; pharmacology_5_; translational medical research_6_; montelukast_7_**

**Supplementary table 2**. Operative definition of comorbidities.

| **Danish Population** | | |
| --- | --- | --- |
| **Comorbidity** | **Definition** | **ICD10^†^ codes** |
| Chronic kidney disease | Defined from diagnosis | E102, E112, E132, E142, I120, M3215, M300, M313, M319, N02-N08, N11-N12, N14, N158-N160, N162-N164, N168, N18-N19, N26, Q612-Q613, Q615, Q619 |
| Heart failure | Defined from diagnosis | *ICD10*^†^: I110, I42, I50, J819 |
| **Italian Population** | | |
| **Comorbidity** | **Definition** | **ICD9^‡^ codes** |
| Chronic kidney disease | Defined from diagnosis | 250.4x, 274.10, 403.xx, 404.xx, 440.1, 442.1, 453.3, 581.xx, 582-583, 585-587, 593.xx, 599.7, 753.0, 753.3, 791.0, 791.2, 866.xx |
| Heart failure | Defined from diagnosis | 428 |

**^†^**ICD10: 10^th^ revision of the International Classification of Diseases system
^‡^ICD9: 9^th^ revision of the International Classification of Diseases system
