# Supplementary material for: Relationship Between Gender and the Effectiveness of Montelukast: An Italian/Danish Register-Based Retrospective Cohort Study
Source: Front Pharmacol. 2018 Aug 2;9:844. doi: 10.3389/fphar.2018.00844 (PMC6083053; doi:10.3389/fphar.2018.00844)
Supplement: Supplementary file 3 [file Table_3.DOCX]

Supplementary Material

Relationship between gender and the effectiveness of montelukast: an Italian/Danish register-based retrospective cohort study

Maurizio Sessa^1,2^⸸ & Annamaria Mascolo^2^⸸, Bruno D’Agostino^2^, Antonio Casciotta^3^, Vincenzo D’Agostino^3^, Fausto De Michele^4^, Mario Polverino^5^, Giuseppe Spaziano^2^, Mikkel Porsborg Andersen^6^, Kristian Kragholm^6^, Francesco Rossi^2^, Christian Torp-Pedersen^6,7^ and Annalisa Capuano^2^.

^1^Department of Drug Design and Pharmacology, University of Copenhagen, Copenhagen, Denmark
^2^Department of Experimental Medicine, University of Campania “L. Vanvitelli”, Naples, Italy.
^3^Pharmaceutical Department, Local Health Unit Napoli Second, Napoli, Italy.
^4^Department of Pneumology, AORN A. Cardarelli, Naples, Italy.
^5^Department of Pneumology and Endoscopic Unit, Ospedale Scarlato, Scafati, Italy.
^6^Unit of Epidemiology and Biostatistics, Aalborg University Hospital, Aalborg, Denmark
^7^Department of Health Science and Technology, Aalborg University, Aalborg, Denmark.

⸸ These authors contributed equally and served as co-first authors.

*** Correspondence:**Maurizio Sessa
maurizio.sessa@sund.ku.dk

**Keywords: clinical epidemiology_1_; asthma_2_; humans_3_; pharmacoepidemiology_4_; pharmacology_5_; translational medical research_6_; montelukast_7_**

**Supplementary table 3**. Pharmacological treatments considered as study covariates for statistical analyses.

| **Drugs** | **ATC^†^ code** |
| --- | --- |
| Hormonal contraceptives | G03A, G03B, G03D, G02B |
| Testosterone-5-alpha reductase inhibitors | G04CB |
| Drugs with recognized pharmacokinetics drug-drug interactions with montelukast | J05AX16, J05AX66, L01DB11, C10AB04, C07AA05, C07FX01, C07AB05, L02BX03, L01XX35, J01EE02, J01EE05, J01EE03, J01EE04, J01EA01, J01EE07, N03AA02, J04AB02, J04AM02, J04AM05, J04AM06 |
| Antipsychotics | N05A |
| Beta-blockers | C07AG02 C07AB02, C07FB02, C07CB02, C07BB02, C07BB52, C07AB52 C07AB07, C07FB07, C07BB07, C07AB57, C09BX02 C07AB12, C07FB12, C07BB12 |
| Nonsteroidal anti-inflammatory drug | M01A, M02B |
| Low-dose acetylsalicylic acid | B01AC06 |
| Antibacterials for systemic use | J01 |
| Selective beta-2-adrenoreceptor agonists inhalants – short acting | R03AC04, R03AC05, R03AC03, R03AC02 |
| Selective beta-2-adrenoreceptor agonists oral – short acting | R03CC03, R03CC02 |
| Selective beta-2-adrenoreceptor agonists inhalants – long acting | R03AC18, R03AC13, R03AC12 |
| Selective beta-2-adrenoreceptor agonists oral – long acting | R03CC12 |
| Glucocorticoids | R03BA01, R03BA05, R03BA02, R03BA08, R03BA03 |
| Mast cell stabilizer | R01AC07, R03BC03, R03BC01, R01AC01 |
| Glucocorticoids + Selective beta-2-adrenoreceptor agonists inhalants | R03AK07, R03AK06 |
| Mast cell stabilizer + Selective beta-2-adrenoreceptor agonists inhalants | R03AK04, R01AC51 |

^†^ATC: Anatomical Therapeutic Classification
